# Supplementary material for: Efficacy of cancer-specific anti-podoplanin CAR-T cells and oncolytic herpes virus G47Δ combination therapy against glioblastoma
Source: Mol Ther Oncolytics. 2022 Jul 20;26:265–74. doi: 10.1016/j.omto.2022.07.006 (PMC9364057; doi:10.1016/j.omto.2022.07.006)
Supplement: Document S1. Figure S1 [file mmc1.pdf]

OMTO, Volume *26*

Supplemental information

Efficacy of cancer-specific anti-podoplanin CAR-T cells and oncolytic herpes virus G47Δ combination therapy against glioblastoma

Lushun Chalise, Akira Kato, Masasuke Ohno, Sachi Maeda, Akane Yamamichi, Shunichiro Kuramitsu, Satoshi Shiina, Hiromi Takahashi, Sachiko Ozone, Junya Yamaguchi, Yukinari Kato, Yumi Rockenbach, Atsushi Natsume, and Tomoki Todo

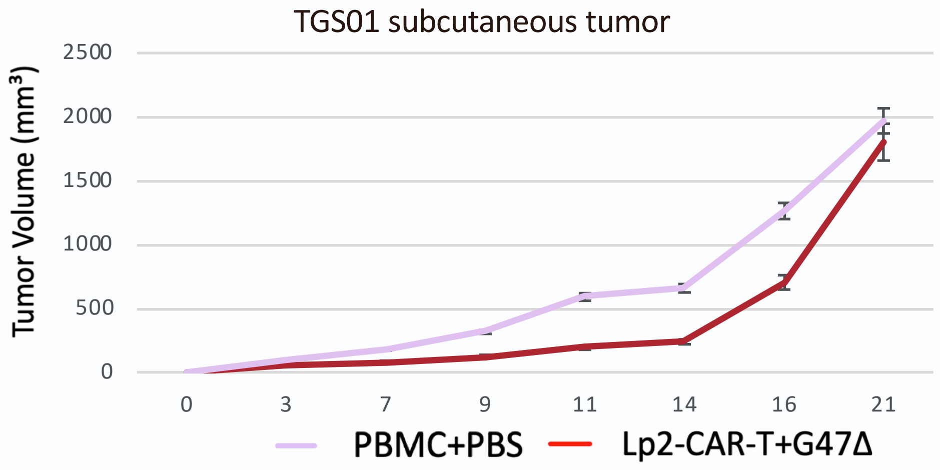

Figure S1. Against TGS01 subcutaneous tumors, the combination of Lp2-CAR-T cells and G47Δ slowed tumor growth for a short period, and it did not improve overall survival
