# Supplementary material for: Efficacy of cancer-specific anti-podoplanin CAR-T cells and oncolytic herpes virus G47Δ combination therapy against glioblastoma
Source: Mol Ther Oncolytics. 2022 Jul 20;26:265–74. doi: 10.1016/j.omto.2022.07.006 (PMC9364057; doi:10.1016/j.omto.2022.07.006)
Supplement: Document S2. Article plus supplemental information [file mmc2.pdf]

Molecular Therapy
Oncolytics
Original Article

Efficacy of cancer-specific anti-podoplanin CAR-T cells and oncolytic herpes virus G47Δ combination therapy against glioblastoma

Lushun Chalise,[1,2,3] Akira Kato,[11] Masasuke Ohno,[4] Sachi Maeda,[1] Akane Yamamichi,[5] Shunichiro Kuramitsu,[6] Satoshi Shiina,[7] Hiromi Takahashi,[8] Sachiko Ozone,[11] Junya Yamaguchi,[1] Yukinari Kato,[9,10] Yumi Rockenbach,[11] Atsushi Natsume,[11,12] and Tomoki Todo[3]

[1]Department of Neurosurgery, Nagoya University School of Medicine, Nagoya, Japan; [2]Department of Neurosurgery, Nagoya Central Hospital, Nagoya, Japan; [3]Division of Innovative Cancer Therapy, Advanced Clinical Research Center, Institute of Medical Science, The University of Tokyo, Tokyo, Japan; [4]Department of Neurosurgery, Aichi Cancer Centre Hospital, Nagoya, Japan; [5]Department of Neurological Surgery, University of California San Francisco, San Francisco, CA, USA; [6]Department of Neurosurgery, National Hospital Organization Nagoya Medical Center, Nagoya, Japan; [7]East Nagoya Imaging Diagnosis Center, Nagoya, Japan; [8]Department of Biomolecular Engineering, Graduate School of Engineering, Nagoya University, Nagoya, Japan; [9]Department of Molecular Pharmacology, Tohoku University Graduate School of Medicine, Sendai, Japan; [10]Department of Antibody Drug Development, Tohoku University Graduate School of Medicine, Sendai, Japan; [11]The Institute of Innovation for Future Society, Nagoya University, Nagoya, Japan; [12]Department of Neurosugery, Kawamura Medical Society Hospital, Gifu, Japan

Glioblastoma is a devastating malignant brain tumor with a poor prognosis despite standard therapy. Podoplanin (PDPN), a type I transmembrane mucin-like glycoprotein that is overexpressed in various cancers, is a potential therapeutic target for the treatment of glioblastoma. We previously reported the efficacy of chimeric antigen receptor (CAR)-T cells using an anti-pan-PDPN monoclonal antibody (mAb; NZ-1)-based third-generation CAR in a xenograft mouse model. However, NZ-1 also reacted with PDPN-expressing normal cells, such as lymphatic endothelial cells, pulmonary alveolar type I cells, and podocytes. To overcome possible on-target-off-tumor effects, we produced a cancer-specific mAb (CasMab, LpMab-2)-based CAR. LpMab-2 (Lp2) reacted with PDPN-expressing cancer cells but not with normal cells. In this study, Lp2-CAR-transduced T cells (Lp2-CAR-T) specifically targeted PDPN-expressing glioma cells while sparing the PDPN-expressing normal cells. Lp2-CAR-T also killed patient-derived glioma stem cells, demonstrating its clinical potential against glioblastoma. Systemic injection of Lp2-CAR-T cells inhibited the growth of a subcutaneous glioma xenograft model in immunodeficient mice. Combination therapy with Lp2-CAR-T and oncolytic virus G47Δ, a third-generation recombinant herpes simplex virus (HSV)-1, further inhibited the tumor growth and improved survival. These findings indicate that the combination therapy of Lp2-CAR-T cells and G47Δ may be a promising approach to treat glioblastoma.

INTRODUCTION

Glioblastoma (GBM) is a devastating malignant brain tumor with a median survival of less than 2 years despite standard therapy that includes surgery followed by chemotherapy and radiation.[1,2] Immunotherapy, the fourth modality of anti-cancer treatment following surgery, chemotherapy, and radiotherapy, has shown promising results in some cancer types but does not have the same success in the treatment of brain tumors. This calls for innovative measures to make immunotherapy more effective against brain tumors. Chimeric antigen receptor (CAR) T cells, which have already proven their clinical efficacy in patients with CD19-positive hematological tumors, such as leukemia[3,4] and lymphoma,[5-7] have so far been disappointing in the fight against brain tumors, with very limited success at the cost of severe adverse events, such as cytokine storm, pulmonary toxicity, and edema, resulting in multiple organ dysfunction, possibly due to on-target-off-tumor effects.[8-11] Therefore, new measures to overcome potential side effects while increasing antitumor efficacy need to be developed.

Podoplanin (PDPN), a type I transmembrane mucin-like glycoprotein, is abundant in several solid tumors including squamous cell carcinoma, malignant mesothelioma, Kaposi sarcoma, angiosarcoma, testicular seminoma, and brain tumors.[12,13] In gliomas, PDPN is overexpressed in accordance with tumor malignancy[14] and is the highest in the mesenchymal subtype GBM.[12,15,16] The potential of PDPN has been recognized as a therapeutic target of CAR T cells to treat brain tumors, and we previously generated a third generation

Received 23 December 2021; accepted 15 July 2022;
https://doi.org/10.1016/j.omto.2022.07.006.

Correspondence: Tomoki Todo, MD, PhD, Division of Innovative Cancer Therapy, Advanced Clinical Research Center, Institute of Medical Science, The University of Tokyo, 4-6-1 Shirokanedai, Minato-ku, Tokyo 108-8639, Japan.
E-mail: atsushi.natsume@mirai.nagoya-u.ac.jp
Correspondence: Atsushi Natsume, MD, PhD, The Institute of Innovation for Future Society, Nagoya University, NIC Room 803, Furo-Cho, Chikusa-Ku, Nagoya 464-8601, Japan.
E-mail: toudou-nsu@umin.ac.jp

This is an open access article under the CC BY license (http://creativecommons.org/licenses/by/4.0/).

of CAR that targeted PDPN using an anti-pan-PDPN antibody, NZ-1-based single-chain variable fragments (scFvs).[12,17] NZ-1-CAR T cells showed specific efficacy against PDPN-positive GBM cells *in vitro* and inhibited the growth of intracranial glioma xenografts *in vivo*.[12] However, PDPN is also present in normal tissues, such as the mesothelium, the lymphatic endothelium, lung type I alveolar cells, kidney glomerular podocytes, meninges,[18,19] and Purkinje cells[20] in the central nervous system. This raises safety concerns regarding PDPN-targeting therapies and necessitates the development of a better approach to specifically target cancer cells to overcome the on-target-off-tumor effect. To precisely target GBM cells, we previously created a cancer-specific monoclonal antibody (CasMab) for human PDPN.[21] A novel monoclonal antibody (mAb) clone, LpMab-2 (Lp2), recognizes the aberrantly glycosylated cancer-type PDPN, which was extracted from a human PDPN-transduced GBM cell line. As Lp2 does not recognize PDPN-expressing normal cells, CAR-T cell therapy based on Lp2 can also be expected to be cancer specific.

Oncolytic viruses are promising anti-cancer therapies that have also been tested against malignant brain tumors.[22-24] They can not only kill solid tumors directly but also elicit an antitumor immune response.[25,26] G47Δ is a third-generation oncolytic recombinant herpes simplex virus (HSV)-1 that was developed by adding another mutation to the genome of G207, a second-generation HSV-1.[27,28] In addition to mutations in the *γ34.5* and *ICP6* genes, G47Δ has a deletion in the *α47* gene that causes enhanced stimulation of antitumor immunity and improved replication in cancer cells. G47Δ proved its efficacy and safety in a phase II clinical trial for GBM in Japan and was approved by the Ministry of Health, Labour and Welfare Japan as the first oncolytic therapy drug for malignant glioma in the world in June 2021.

Given the fact that G47Δ induces a specific antitumor immune response, a combination of CasMab-based CAR-T cells may exert a more rational antitumor therapy specific to GBM.

In this study, we established a third generation of Lp2 scFv-based CAR that reacts with cancer-type PDPN in GBM. We report an enhanced antitumor effect when this novel CAR-T cell was used in combination with the oncolytic virus G47Δ.

RESULTS

Cancer-type PDPN expression in cell lines

Immunofluorescence using the antibody Lp2 demonstrated that cancer-type PDPN was positive on the cell surface of LN319, GIC0222, and TGS01 cells. LN229 cells did not express PDPN and were therefore used as a negative control. LN229 cells transfected with human PDPN were used as positive controls for subsequent *in vitro* experiments (Figure 1A). Normal mesothelial cells, MeT-5A expressing PDPN were positive for anti-pan-PDPN mAb NZ-1, but negative for the CasMab Lp2.[21] MeT-5A cells were later used in an *in vitro* cytotoxicity assay to confirm the cancer specificity of CAR-T cells based on Lp2.

Construction of Lp2-CAR-T cells

A CasMab, Lp2 was purified from a human PDPN-transfected GBM cell line and recognizes cancer-type PDPN.[21] We constructed a lentiviral vector linked with the EF1α promoter tandemly followed by the leader sequence and Lp2-based scFvs, CD28, 4-1BB, and CD3ζ (third-generation CAR) (Figure 1B). The transduction efficiency was examined by flow cytometry using a mouse-derived $F(ab')_2$-biotin antibody. The percentage of transduced peripheral blood mononuclear cells (PBMCs) with Lp2-CAR was more than 40%, and these results were reproducible (Table 1). This novel CAR-T (called Lp2-CAR-T) based on CasMab is expected to exhibit cancer-specific cytotoxicity against glioma.

In vitro functional assay of Lp2-CAR-T cells

The calcein-based non-radioisotope cytotoxic assay demonstrated that Lp2-CAR-T cells significantly lysed PDPN-positive LN229/human PDPN (hPDPN) cells but not PDPN-negative LN229 cells and Met-5A cells. Lp2-CAR-T cells showed significant dose-dependent cytolysis (effector/target ratio >6) in LN229/hPDPN cells, whereas no significant difference in cytolysis was observed in PDPN-negative LN229 cells with Lp2-CAR-T and PBMCs (Figure 2A). Mock CAR-T cells were CAR-T cells without a scFv portion. We found that mock CAR-T cells did not show any specific cytolysis of hPDPN-transfected LN229 cells.

Interferon (IFN)-γ production was measured after the co-culture of different cell lines with Lp2-CAR-T cells at an effector/target ratio of 20 for 24 h (Figure 2B). IFN-γ release in the supernatant of LN229/hPDPN cells co-cultured with Lp2-CAR-T cells produced 100.1 pg/mL of IFN-γ, which was more than 10-fold higher than that with mock CAR-T cells (9.6 pg/mL; $p < 0.05$). IFN-γ release after the co-culture of PDPN-negative LN229 cells with both Lp2-CAR-T and mock CAR-T cells was 10.9 and 12.5 pg/mL, respectively. Co-culture of TGS01 cells with Lp2-CAR-T produced 95.5 pg/mL IFN-γ, whereas mock CAR-T cells released only 7.8 pg/mL IFN-γ. The production of IFN-γ under the co-culture of MeT-5A cells with Lp2-CAR-T and mock CAR-T was undetectable. There was no difference in the on-target effect between mock CAR-T cells and PBMCs, and PBMCs were used as a control effector in the following experiments.

Real-time cell analysis showed that the percentages of lysis of the cancer-type PDPN-expressing glioma cells LN229/hPDPN and LN319 were significantly higher with Lp2-CAR-T cells than with PBMCs. This cytolytic effect was obvious after 3 h of co-culture and was observed continuously for 15 h (Figure 2C). However, Lp2-CAR-T cells did not show any specific cytolysis of LN229 (GBM cells that do not express PDPN). We also observed the cytolytic effect of Lp2-CAR-T cells on patient-derived glioma stem cells, GIC0222 and TGS01, both of which express cancer-type PDPN. Lp2-CAR-T cells lysed both GIC0222 and TGS01 cells (Figure 2C).

Efficacy of Lp2-CAR-T cells in combination with oncolytic virus G47Δ *in vitro*

The co-culture of PDPN-transfected LN229/hPDPN cells and Lp2-CAR-T cells produced 100.1 pg/mL IFN-γ, whereas PBMCs

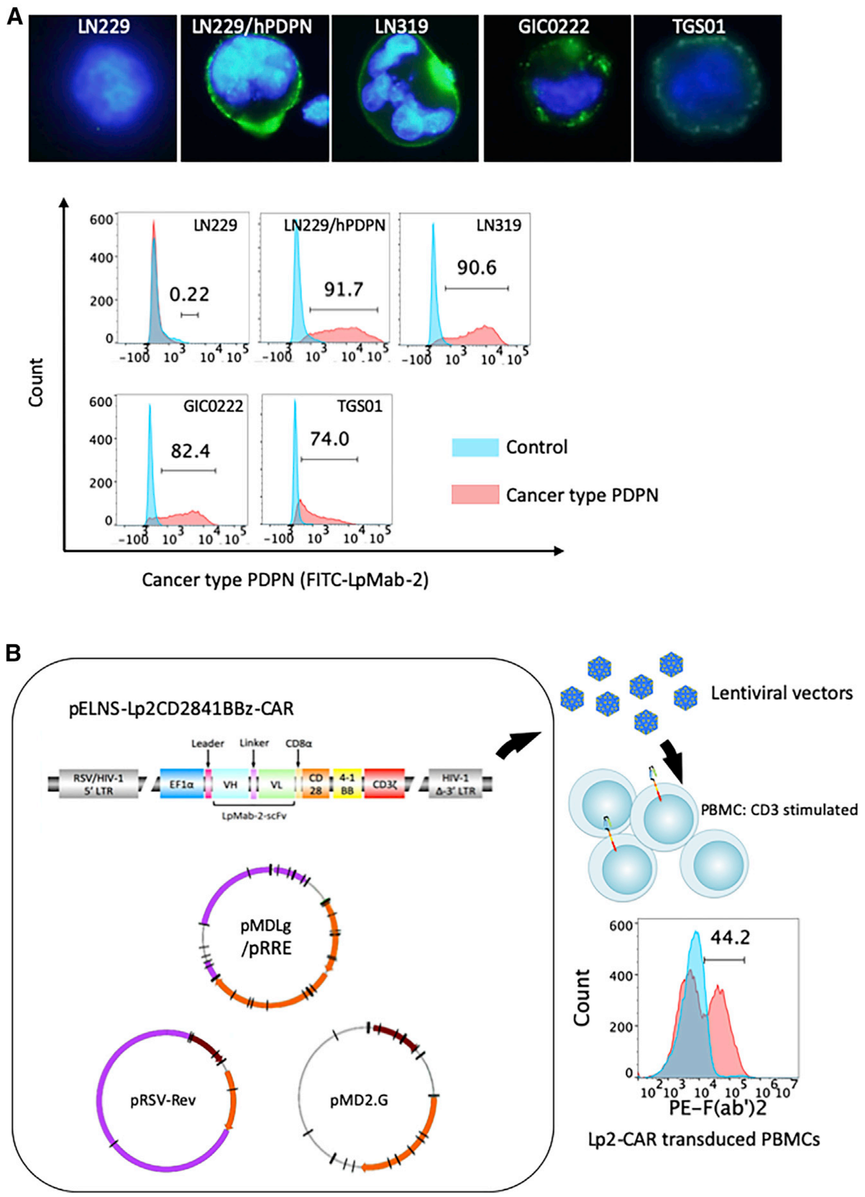

Figure 1. Expression of cancer type podoplanin in glioblastoma, and construction of Lp2-chimeric antigen receptor-T cells

(A) Expression of cancer type podoplanin (PDPN) in glioblastoma (GBM) cell lines. Immunostaining (top, immunofluorescence; bottom, flow cytometry) using cancer-specific anti-PDPN LpMab-2 (Lp2) against PDPN-positive cancer cells (LN229/hPDPN), PDPN-negative cancer cells (LN229), and PDPN-positive normal cells (HEK293T). LN319 and two patient-derived glioma stem cell lines established at our institutions (GIC0222 and TGS01) showed cancer-type PDPN expression as detected by Lp-2. Immunofluorescence for PDPN (green) is consistent with the results of flow cytometry. (B) Construction of Lp2-chimeric antigen receptor (CAR)-T cells. Lentiviral vector construct with the EF1α promoter followed by the leader sequence, Lp-2-based single-chain variable fragments (scFvs), CD28, 4-1BB, and CD3ζ. The transduction efficiency, examined by flow cytometry with mouse-derived F(ab')[2] recognizing biotin antibody, was 44.2% and reproducible (see also Table 1).

released 9.6 pg/mL IFN-γ. When G47Δ, at a multiplicity of infection (MOI) of 0.1, was added to Lp2-CAR-T cells, IFN-γ production increased to 152.9 pg/mL. IFN-γ was not increased when G47Δ was added to cultures of Lp2-CAR T and PDPN-negative LN229 cells. The addition of G47Δ increased IFN-γ production from 67.1 to 98.3 pg/mL in the co-culture of TGS01 and Lp2-CAR-T cells. An increase in IFN-γ release was not observed when G47Δ was added to PBMCs in LN229/hPDPN, LN229 and TGS01 cells (Figure 3A). A real-time cell proliferation assay using iCELLigence showed that either Lp2-CAR-T cells or G47Δ alone suppressed cell proliferation significantly, and the combination further decreased cell viability in both LN229/hPDPN and TGS01 (Figure 3B). These results showed that the combination of Lp2-CAR-T and G47Δ was more effective than Lp2-CAR-T alone *in vitro*. Moreover, the antitumor effect was observed earlier when Lp2-CAR T and G47Δ were used in a combination therapy compared with any other monotherapy.

Efficacy of Lp2-CAR-T cells on GBM in the mouse subcutaneous xenograft

Subcutaneous tumors were established by injecting the mice with PDPN-transfected LN229/hPDPN glioma cells, and treatment was started when the mean tumor diameter reached 5 mm. G47Δ was injected into the tumor, and CAR-T cells were injected intravenously via the retroorbital vein. The treatment design is summarized in Figure 4A. Both G47Δ and Lp2-CAR-T inhibited tumor growth significantly compared with the control (PBMCs and phosphate-buffered saline [PBS]). However, combination therapy significantly inhibited tumor growth (Figure 4B). The mean tumor volume of all four groups at day 28 of treatment showed that mice treated with mock CAR-T cells and mock virus had significantly larger tumor size compared with both Lp2-CAR-T cell therapy and G47Δ monotherapy ($p < 0.05$) and combination therapy ($p < 0.005$). Moreover, the combination therapy significantly inhibited tumor growth compared with monotherapy ($p < 0.05$).

The survival curves of the four groups are shown in Figure 4C. A log rank test indicated that while G47Δ- and Lp2-CAR-T-treated mice survived longer than control mice, the combination therapy prolonged survival significantly, and half the mice survived longer, for

Table 1. Transduction efficiency of Lp2-CAR T

Lp2-CAR-T (batch number)	Transduction efficiency (%)
1	68.7
2	44.2
3	40.6
4	41
5	65.9
Median	44.2

more than 60 days. The median overall survival (OS) durations of the four groups were as follows: mock CAR-T (33 days), G47Δ (35 days), Lp2-CAR-T (42 days), and combination of G47Δ and Lp2-CAR-T (51 days) ($p = 0.002$). In all mice, the cause of death was tumor burden, and no side effects of CAR-T treatment were observed in the long-term survivors in the combination group.

Against TGS01 subcutaneous tumors, the combination of Lp2-CAR-T cells and G47Δ slowed tumor growth for a short period, and it did not improve OS (Figure S1).

DISCUSSION

We constructed a novel CasMab-based CAR T cells that targets the cancer-specific PDPN in GBM cells in this study. We successfully transduced cancer-specific CAR on human T cells by lentivirus-mediated expression and demonstrated that the engineered T cells were specific and effective against cancer-specific PDPN-positive cells. These CAR-T cells showed specific cytolysis against GBM cells *in vitro*, and systemic injection of these CAR-T cells significantly inhibited the tumor growth *in vivo* ($p < 0.05$). Several CAR-T cells have been developed to target GBM, and a few, including epidermal growth factor receptor (EGFR)-VIII, human epidermal growth factor receptor 2 (HER2), and interleukin (IL)-13Rα2-targeting CAR-T cells, have been applied in clinical trials with promising results in a limited number of patients.[29-33] These mixed results point toward the fact that while CAR-T cell therapy is indeed effective against brain tumors, it does have its own limitations. Overcoming these limitations related to the safety and efficacy of CAR-T cells holds the key to improving the therapy against brain tumors.

We have previously reported the construction and efficacy of NZ-1-CAR-T cells that target PDPN;[12] however, NZ-1 CAR-T cells may have an on-target-off-tumor effect because they may recognize low levels of PDPN even in normal tissues. The on-target-off-tumor effect is a well-known adverse event of CAR-T cell therapy and can have devastating effects on patients, even death.[10,34-36] Lp2 recognizes cancer-type PDPN, which is extraordinarily glycosylated.[21] Lp2 can react with PDPN-expressing cancer cells but not with normal cells, such as mesothelium. Therefore, we produced a new CAR using Lp-2 to overcome the on-target-off-tumor phenomenon. We demonstrated that this novel CAR-T cell was effective against glioma cells that express PDPN but did not have any cytolytic effect against PDPN-expressing non-cancer cells like mesothelial cells. CasMab-based CAR-T therapies may improve the safety and specificity to target tumors, while the expression of cancer-specific antigen in the tumor and normal tissues should be elaborately tested in the next preclinical trials.

Although Lp2-CAR-T monotherapy was enough to moderately inhibit tumor growth, addition of the oncolytic virus G47Δ to CAR-T cell therapy drastically improved the antitumor efficacy. Oncolytic viruses not only cause direct tumor killing but may also initiate antitumor immune reactions, making the tumor microenvironment favorable for CAR-T infiltration. Intriguingly, the combination of G47Δ with Lp2-CAR-T cells elicited greater release of IFN-γ and had a greater killing effect on PDPN-expressing GBM cells even though the combination of G47Δ and non-specific T cells did not. G47Δ may bolster the on-target effect of specific T cells. Several attempts have been made to enhance the efficacy of CAR-T cells against solid tumors by adding an oncolytic virus to therapeutic strategies.[37-39] Oncolytic viruses have also been armed with chemokines[40] and immune checkpoint programmed cell death-ligand 1 (PD-L1) mini antibodies to enhance the efficacy of CAR-T cells against solid tumors.[41] GBM is a heterogeneous tumor that is difficult to treat by targeting a single antigen, and antigen loss after the administration of CAR-T cells is also a major concern.[42] G47Δ targets tumor cells independent of PDPN expression, thus killing tumor cells that evade Lp2-CAR T cells. Increasing the antitumor efficacy of CAR-T cells using another antitumor therapy may also contribute to the increased safety of CAR-T cells. While a patient treated with a substantially high dose of HER2/neu-specific CAR-T cells (1×10^{10} cells) resulted in fatality,[10] subsequent studies utilizing a different HER2/neu-specific CAR at a significantly lower dose have proven to be safe.[11] Therefore, a combination therapy of CAR-T cells with an oncolytic virus may be safer than any other CAR-T cell monotherapy at a higher dose. While we observed significant efficacy against LN229/hPDPN, an artificial GBM cell line expressing hPDPN, the efficacy against the patient-derived tumor cells, i.e., TGS01 cells, lasted only for a short period of time in the animal experiments. CAR-T cells were injected intravenously, and G47Δ was administered twice intratumorally. The dose and time schedule should be optimized in future preclinical studies.

No adverse effects were observed in mice in the animal experiments. However, it should be noted that this CAR is specific to hPDPN, and animal experiments cannot completely simulate its effect on human tissue. Moreover, it should also be noted that this is a limitation of animal experiments in general, and thorough safety precautions should be taken before any clinical application. Although a number of issues, such as the lack of complete response in most of the mouse models and the clarification of the exact immunological mechanisms involved in the combination therapy, still need to be addressed, we have successfully established novel cancer-specific CAR-T cells against a promising tumor antigen and have reported that it can be used in combination with an oncolytic virus against GBM.

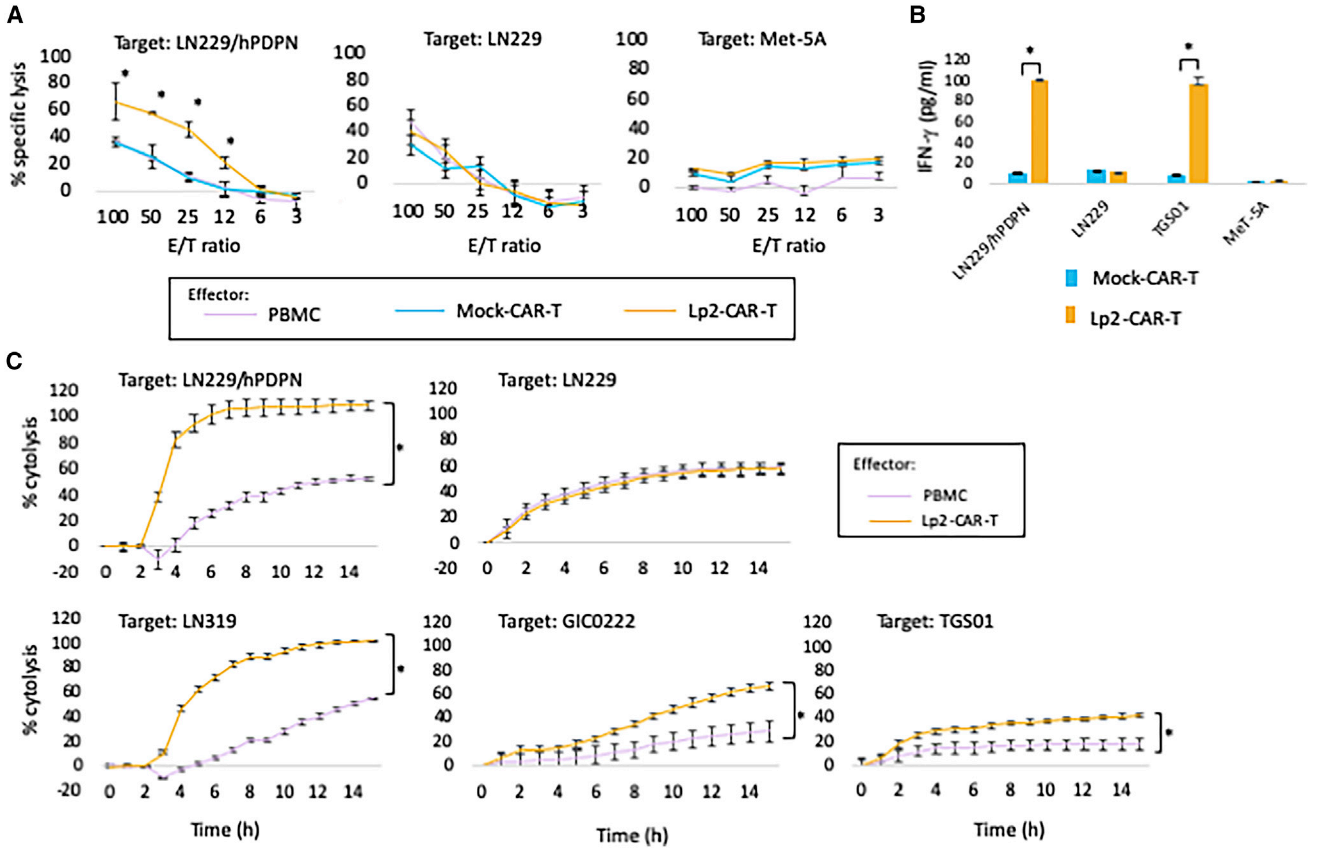

Figure 2. Functional assay of Lp-2-CAR-T cells *in vitro*

(A) PDPN-positive glioma cells (LN229/hPDPN) are lysed significantly by Lp2-CAR–T compared with peripheral blood mononuclear cells (PBMCs) and mock vector-transduced T cells (mock CAR-T) in an effector:target (E:T) ratio-dependent manner; however, specific lysis was not observed in PDPN-deficient glioma cells. Mock versus Lp2-CAR-T, $p < 0.05$ for ET ratio of 12 or more. Means and standard deviation (SD) of 3 wells of the same experiment are shown. (B) Interferon (IFN)-γ release (enzyme-linked immunosorbent assay [ELISA]). The co-culture of LN229/hPDPN or TGS01 with Lp2-CAR-T produced approximately 90–100 pg/mL IFN-γ. The IFN-γ levels were significantly higher than those from mock CAR-T cells ($p < 0.05$). LN229 and MeT-5A cells produced only a similar amount of IFN-γ when co-cultured with both Lp2-CAR-T and mock CAR-T cells. Means and SD are shown. (C) PDPN-expressing glioma cells (LN229/hPDPN and LN319) showed significantly higher cytolysis by Lp2-CAR-T when compared with PBMCs added at the same E:T ratio (6); *$p < 0.05$, for each hour starting at 4 h after addition of CAR-T cells. However, LN229 cells (which do not express PDPN) did not show any specific cytolysis by Lp2-CAR-T when compared with PBMCs. Lp2-CAR-T cells also showed significant cytolysis in patient-derived glioma stem cells, GIC0222 (*$p < 0.05$ after 5 h) and TGS01 (*$p < 0.05$ after 4 h). Both stem cells express PDPN. The means and standard error of the mean (SEM) of three wells of the same experiment are shown.

This is the first study to report a CasMab-based CAR-T cell and demonstrate the efficacy of a combination therapy using CAR-T cells and the oncolytic virus G47Δ against GBM. Since G47Δ has been approved as the first oncolytic virus drug for malignant glioma in the world, the combination therapy with G47Δ and CAR-T cells may be an effective treatment approach for GBM. Despite its limitations, this study provides new insights into a combination therapy of CAR-T cells and oncolytic viruses to treat solid tumors, especially malignant brain tumors that do not respond to the current standard therapies.

MATERIALS AND METHODS

Cell lines

The human GBM cell lines (LN229, LN229/hPDPN, LN319) were maintained in Dulbecco's modified Eagle's medium (DMEM; Sigma-Aldrich, St. Louis, MO, USA) containing 10% heat-inactivated fetal bovine serum (FBS; Thermo Fisher Scientific, Waltham, MA, USA) at 37°C in a humidified atmosphere of 5% carbon dioxide (CO_2). LN319 cells were purchased from Addexbio Technologies (San Diego, CA, USA). LN229 cells were purchased from the American Type Culture Collection (ATCC, Manassas, VA, USA) and transfected with hPDPN to generate LN229/hPDPN cells. Normal mesothelial cells, MeT-5A cells, were also purchased from ATCC. The expression of hPDPN and cancer-specific PDPN on LN229/hPDPN and Met-5A was previously described.[21] The cell lines were authenticated by letters when they were provided. The GBM-initiating cells, GIC0222, were cultured in a neurobasal medium (Thermo Fisher Scientific) supplemented with 2 mmol/L L-glutamine (Sigma-Aldrich, St. Louis, MO, USA), N-2 and B-27

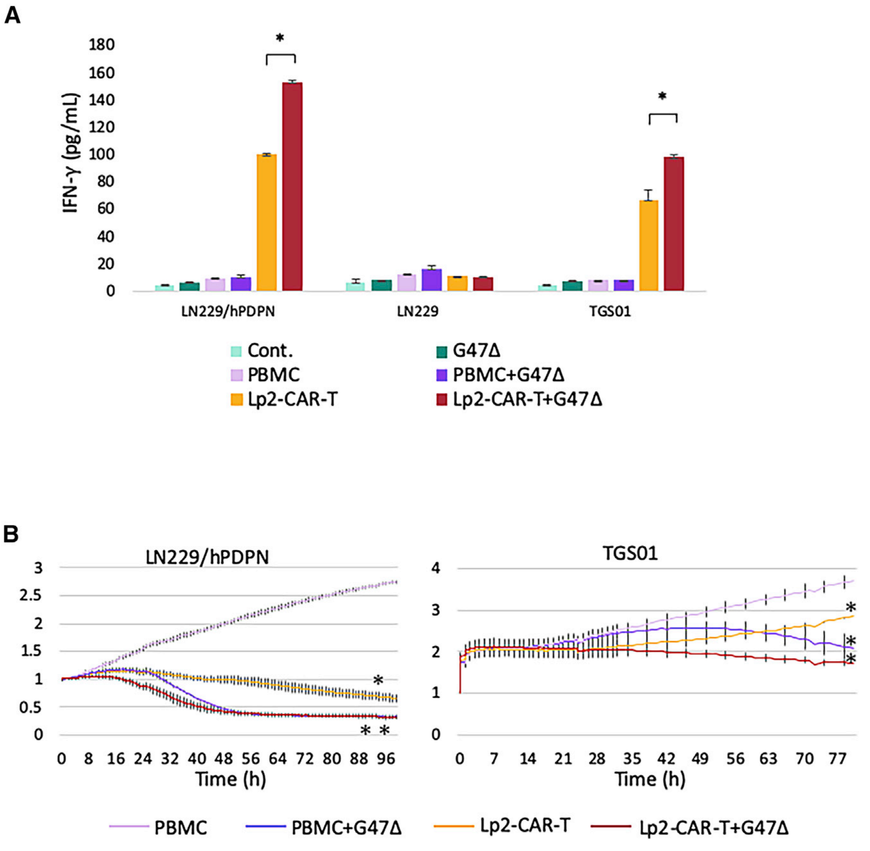

Figure 3. Addition of the oncolytic virus G47Δ to a culture of Lp2-CAR-T cells

(A) The combination against PDPN-expressing glioma cells increased IFN-γ release. When G47Δ, at a multiplicity of infection (MOI) of 0.1, was added to Lp2-CAR-T cells, IFN-γ production in the co-culture of PDPN-transfected LN229 cells (LN229/hPDPN) or TGS01 cells was increased significantly. There was no difference in LN229 cells. IFN-γ was not increased when G47Δ was added to PBMCs. Means and SD are shown. $*p < 0.05$.
(B) Lp2-CAR-T cells in combination with G47Δ inhibit tumor growth *in vitro*. A real-time cell proliferation assay showed a gradual decrease in LN229/hPDPN cells and TGS01 when co-cultured with Lp2-CAR-T cells. Addition of G47Δ to the co-culture further decreased the cell viability. Means and SD are shown. $*p < 0.05$, $**p < 0.01$ compared with PBMCs.

supplements (Thermo Fisher Scientific), recombinant human basic fibroblast growth factor (bFGF) and EGF (16.7 ng/mL each; R&D Systems, Minneapolis, MN, USA), 100 units/mL penicillin, and 100 mg/mL streptomycin (Thermo Fisher Scientific) at 37°C in a humidified atmosphere of 5% CO_2. Glioma-derived cancer stem cells, TGS01, were cultured in DMEM/F12 medium (Thermo Fisher Scientific) supplemented with 200 mM L-glutamine (Sigma-Aldrich), 45% D-glucose solution (Sigma-Aldrich), B-27 supplements (GIBCO, Thermo Fisher Scientific), recombinant human bFGF, and EGF (20 ng/mL each; Peprotech). GIC0222 and TGS01 were primary cultured GBM cells from the GBM tissue sample taken after obtaining written informed consent from all patients undergoing surgery at the Nagoya University Hospital and the Tokyo University Hospital, Japan, respectively. This study was approved by the two institutional review boards. The dissociation procedures have been described elsewhere. All cell lines were tested for the presence of mycoplasma contamination.

PDPN immunofluorescent staining

GBM, GIC0222, and TGS01 cell lines were plated on a 24-well plate containing BD BioCoat poly-L-lysine cellware 12-mm round coverslips with 10% FBS/DMEM and incubated for 24 h.

The coverslips were rinsed twice with phosphate-buffered saline (Thermo Fisher Scientific) and placed in 4% paraformaldehyde phosphate buffer solution (PFA; FUJIFILM Wako Pure Chemical Corporation, Osaka, Japan) for 15 min.

Blocking was performed in PBS containing 0.1% Triton X-100 (PBST; Sigma-Aldrich) with 1.5% goat serum for 1 h at room temperature with shaking. The coverslips were incubated with the primary antibody Lp2, diluted to 1 mg/mL in blocking solution for 1 h at room temperature with shaking, followed by three washes with PBS. The coverslips were stained using a secondary antibody, Alexa Fluor 488 Goat Anti-Rat IgG (HþL; Thermo Fisher Scientific), and 4′,6-diamidino-2-phenylindole dihydrochloride (DAPI) solution (Dojindo, Kumamoto, Japan) at 1:200 dilution in the blocking solution for 30 min in the dark, followed by three washes with PBST. They were then mounted onto slides. Stained cells were observed under a fluorescence microscope (Keyence BZ-X710, Osaka, Japan), and pictures were taken.

Construction of a self-inactivating (SIN) lentiviral vector

The mAb Lp2 has been previously established.[21] The scFv was then produced, and the scFv portion in the previous CAR construct, pELNS-empty-CAR,[43] was changed to the Lp2-based scFv to generate pELNSLp2-CAR by gene synthesis (Genscript Japan, Tokyo, Japan).[12] In this construct, the EF1α promoter drives the CAR fusion protein containing the Lp2-based scFv targeting PDPN, CD28, 4-1BB, and CD3ζ domains.

Preparation of Lp2-CAR T and mock CAR T cells

Lp2 (cancer-specific anti-PDPN)-CAR-T and mock (no scFV) CAR-T cells[43] were prepared by the production and transduction of lentiviral vectors. HEK293T cells (8×10^6) were plated in 175-cm^2 flasks at 37°C in a humidified atmosphere of 5% CO_2. At 24 h, the SIN vector, pMDLg/pRRE, pRSV-Rev, and pMD2.G were co-transfected using the X-tremeGENE 9 DNA transfection reagent (Roche

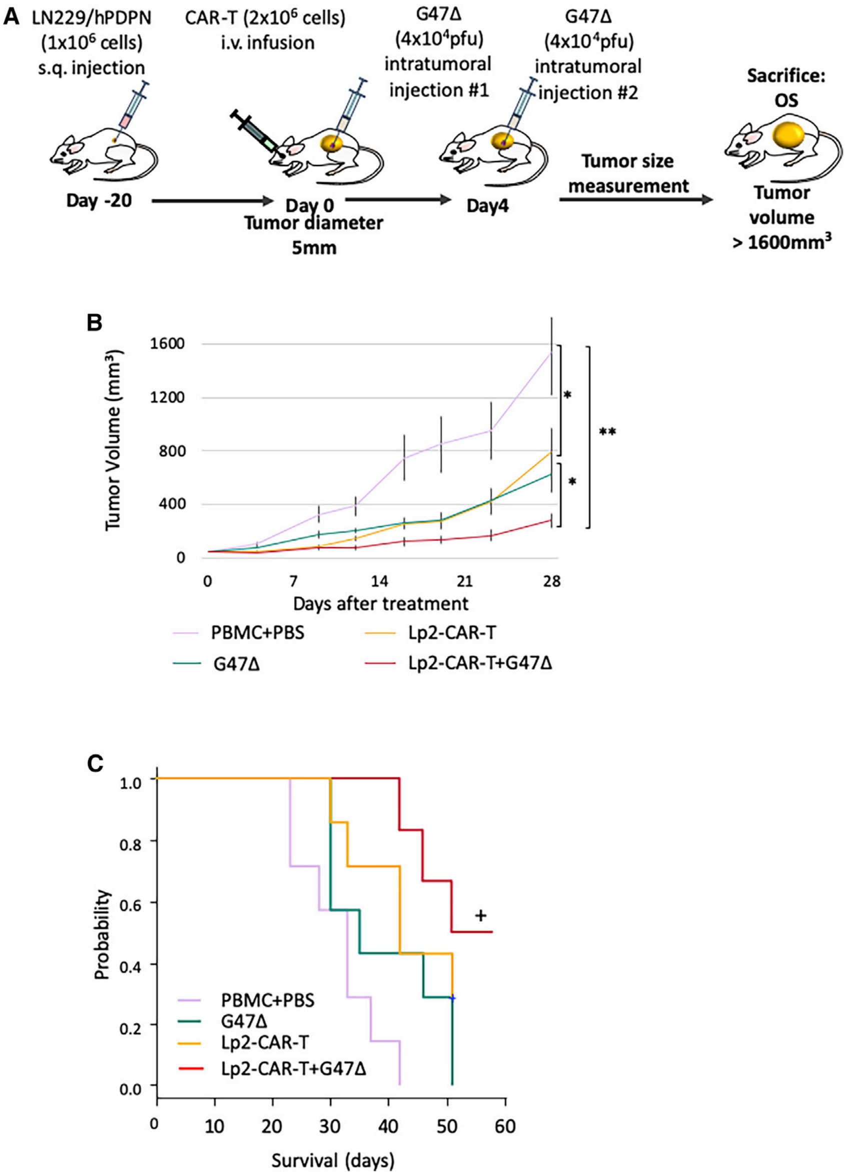

Figure 4. Efficacy of Lp2-CAR-T cells in combination with the oncolytic virus G47Δ *in vivo*

(A) Experimental schema, subcutaneous tumor was established by injecting 6-week-old NOD/Shi-scid, IL2Rr KO Jic (NOG) mice with LN229/hPDPN glioma cells. Treatment was started when tumor diameter reached 5 mm. G47Δ was injected into the tumor, and CAR-T was injected intravenously via the retroorbital vein. On day 4, G47Δ intratumoral injection was repeated. i.v., intravenous injection; OS, overall survival. (B) Tumor volumes determined by caliper measurements. Means and SEM are shown (n = 6 or 7, respectively). Representative of the two experiments. *p < 0.05, **p < 0.005 by two-way analysis of variance (ANOVA). (C) Median overall survival (OS) durations of the four groups (n = 6 or 7) were 33, 35, 42, and 51 days, respectively. p = 0.002 by log rank test. While G47Δ- and Lp2CAR-treated mice survived longer than the control mice, the combination therapy prolonged their survival significantly, and half the mice survived long term.

Applied Science, Penzberg, Germany). The supernatant was collected at 48 h, mixed with PEG-it Virus Precipitation Solution (SBI Funakoshi, Tokyo, Japan), and incubated for 24 h at 4°C. The supernatant/PEG-it mixture was then centrifuged at 1,500 × *g* for 30 min at 4°C. The pellet was resuspended in 1/10 of the original volume using a cold sterile medium at 4°C and was stored at −80°C.

Freshly harvested heparinized PBMCs from healthy donors were separated over a monolayer of Ficoll-Paque PLUS (1,000 × *g* for 20 min at 20°C; GE Healthcare Bio-sciences AB, Uppsala, Sweden). PBMCs were cultured in 4 mL AIM-V medium (Thermo Fisher Scientific) with 2.5% human serum per well of a 6-well plate coated with anti-human CD3 mAbs (Thermo Fisher Scientific) in the presence of IL-2 (50 units/mL; PeproTech) at 37°C in a humidified atmosphere of 5% CO_2 for 24 h. Next, the medium (3 mL/well) was gently removed without disturbing the clustering of PBMCs, and the lentiviral pELNS-Lp2-CAR vector supernatant (X10; 3 mL/well) was added, and the cells were cultured for 24 h. To produce mock CAR-T cells, 3 mL medium was removed in a similar fashion, and 3 mL fresh medium was added to each well, followed by culturing of the cells for 24 h. The medium was then replaced with fresh medium, and the cells were cultured for 48 h. The same batch of PBMCs was used to prepare both mock CAR-T and Lp2-CAR-T cells for each experiment.

Flow cytometric analysis of Lp2-CAR expression

As the construct contained murine-derived fragment antigen-binding region (Fab), Lp2-CAR expression on the cell surface of PBMCs was examined by flow cytometry using either FACSCanto II equipped with the CellQuest Pro software (BD Biosciences, Franklin Lakes, NJ, USA) or Cytoflex (Beckman Coulter, Tokyo, Japan). PBMCs were washed twice with PBS containing 0.5% bovine serum albumin (BSA; Sigma-Aldrich) and 2 mmol/L ethylenediaminetetraacetic acid (EDTA) (Dojindo). PBMCs were then incubated with biotin-AffiniPure F(ab')2 fragment-specific goat anti-mouse IgG (Jackson ImmunoResearch Laboratories, West Grove, PA, USA) at 4°C for 30 min.

After washing twice, PBMCs were stained with streptavidin (SA)-phycoerythrin (PE; BD Biosciences) at 4°C for 30 min in the dark. After washing twice, PBMCs were suspended in 1% PFA and analyzed using flow cytometry Cytoflex. Data were analyzed using FlowJo v.10 (BD Biosciences, Franklin Lakes, NJ, USA).

Analysis of IFN-γ production by effector cells

IFN-γ production by effector cells was measured by enzyme-linked immunosorbent assay (ELISA) using the human IFN-γ ELISA Ready-SET-Go! (Thermo Fisher Scientific). Effector cells (1×10^6 unsorted Lp2-CAR-transduced PBMCs or non-transduced PBMCs) were co-cultured with 1×10^4 target cells (LN229, LN229/hPDPN, TGS01, or MeT-5A) in each well of a 96-well plate for 24 h. The cell culture supernatants were harvested and used for ELISA. Cancer-type PDPN was highly expressed in LN229/hPDPN cells but not in LN229cells. Therefore, these cells were used as the positive and negative controls, respectively. While PDPN is also expressed in MeT-5A cells, it is not a cancer cell; therefore, the non-reactivity of MeT-5A cells to Lp2-CAR-T cells would serve as a confirmation of cancer specificity of Lp2-CAR-T cells.

Cytotoxicity assay

Target cells (LN229, LN229/hPDPN, LN319, Met-5A, GIC0222, and TGS01) were suspended at a final concentration of 1×10^6 cells/mL and incubated with 10 mmol/L (100 times) calcein-AM solution (Dojindo) at 37°C for 30 min with occasional shaking. After washing twice, the cells were adjusted to 1×10^5 cells/mL, and 1×10^4 cells (100 mL) were placed into a well of a round-bottom 96-well plate. Effector cells (NZ-1-CAR-transduced PBMCs, 3C10-CAR-transduced PBMCs, or non-transduced PBMCs) were incubated for 48 h. The cells were then harvested and added to each well at an appropriate effector:target ratio of 50:1, 25:1, 12.5:1, 6:1, and 3:1 and incubated at 37°C for 5 h. After centrifugation at $300 \times g$ for 2 min, 75 mL supernatant was aspirated carefully and loaded into a 96-well white/clear flat-bottom plate (BD Biosciences). The absorbance was then measured to evaluate the tumor-killing efficacy.

Target cells in the medium with 3 mL of 10% sodium dodecyl sulfate (SDS; Wako, Osaka, Japan) were used to determine the maximum release, and target cells alone were used to measure the spontaneous release. The percentage of specific lysis was calculated as follows: 100 × (experimental release−spontaneous release)/(maximum release−spontaneous release).

Cytotoxicity assay using real-time cell analyzer (RTCA)

Kinetic analysis of tumor cell lysis was performed using iCELLigence and xCELLigence RTCA (ACEA Biosciences, San Diego, CA, USA). Twenty thousand tumor cells were seeded onto E-Plate L8 PET (ACEA Biosciences). After 24-h culture, T cells or control media were added. The cell index (CI) was recorded every 1 h, and cytotoxicity was calculated as {CI (control medium)−CI (effector cells)}/CI (control medium).

To analyze the additional effect of adding G47Δ, the tumor cells were first infected with G47Δ after 24 h of culture, and the supernatant was replaced with fresh culture media after 1 h of infection. After another 1 h culture, T cells or control media were added, and CI was recorded every hour.

Subcutaneous glioma xenograft model

Immunofluorescence analysis indicated that PDPN was highly expressed in LN319 cells. However, this cell line is not tumorigenic *in vivo*.[44] Thus, we utilized LN319 cells only in the *in vitro* experiments. LN229/hPDPN cells were found to be tumorigenic in preliminary experiments. Therefore, LN229/hPDPN cells were used in animal experiments. All animal experiments were approved by the ethics committee of The University of Tokyo and were performed in accordance with our institutional animal care and use guidelines. Six-week-old NOD/Shi-scid, IL2Rγ null (NOG) female mice (Central Institute for Experimental Animals, Kawasaki, Japan) were used for the experiments.

Mice were anesthetized intraperitoneally (i.p.) with a solution of ketamine (75 mg/kg body weight) and xylazine (100 mg/kg body weight). After shaving the left flank of the mice, 1×10^6 LN229/hPDPN cells/mouse suspended in 100 μL PBS were injected subcutaneously. When the mean tumor diameter reached 5 mm, mice were randomized and treated with an intratumoral injection of either PBS or 4×10^4 PFU of G47Δ, followed by intravenous injection of either Lp2-CAR-transduced PBMCs or non-transduced PBMCs. PBMCs (2×10^6 cells) were suspended in 200 μL PBS and infused intravenously (i.v.) via the retroorbital vein. Untreated mice were infused with PBS alone. An intratumor injection with either PBS or 4×10^4 PFU of G47Δ was repeated once, 4 days after the first treatment; however, no additional CAR-T cells were administered. Tumor size and survival were monitored following tumor inoculation. Tumor dimensions were measured with a digital caliper, and tumor volumes were calculated as follows: volume = (length × width × height)/2. The mice were euthanized when the tumor length reached 20 mm or when the tumor volume reached 1,600 mm^3.

Statistics

Student's t test was used to determine the statistical significance of differences between the two groups, and a two-tailed p value of <0.05 was considered to be statistically significant. Mouse survival curves were obtained using the Kaplan-Meier method, and the difference between curves was compared using the log rank test with Bonferroni's correction.

DATA AND CODE AVAILABILITY

Experimental data and applicable methodology documents may be available upon request to the corresponding authors (A.N. and T.T.).

SUPPLEMENTAL INFORMATION

Supplemental information can be found online at https://doi.org/10.1016/j.omto.2022.07.006.

ACKNOWLEDGMENTS

T.T. owns the patent for G47Δ in Japan. This work was supported in part by a Grant-in-Aid for Young Scientists (L.C., 19K18416); the Fund for the Promotion of Joint International Research B (A.N., 19KK0228); a Grant-in-Aid for Scientific Research on Innovative Areas (A.N., 17H06356); a Grant-in-Aid for Scientific Research B

(A.N., 20H03789); the Practical Research for Innovative Cancer Control, Japan Agency for Medical Research and Development (T.T., AMED; JP18ck0106416), the Translational Research Program, AMED (T.T., JP20lm0203140); the Science and Technology Platform Program for Advanced Biological Medicine, AMED (Y.K., JP21am0401013); and BINDS, AMED (Y.K., JP21am0101078). We would like to thank Ms. Junna Natsume for her editorial assistance.

AUTHOR CONTRIBUTIONS

Study concept and design, A.N. and T.T.; provision of study materials, Y.K.; data collection and assembly, L.C., A.K., M.O., S.M., A.Y., S.K., S.S., S.O., and J.Y.; data analysis and interpretation, A.N. and T.T.; development of tables and figures, L.C., H.T., Y.R., and A.N.; writing the report, L.C., A.N., and T.T.; critical review of the manuscript and approval of the final version, all authors.

DECLARATION OF INTERESTS

The authors have no conflict of interest to declare.

REFERENCES

1. Stupp, R., Mason, W.P., van den Bent, M.J., Weller, M., Fisher, B., Taphoorn, M.J., Belanger, K., Brandes, A.A., Marosi, C., Bogdahn, U., et al. (2005). Radiotherapy plus concomitant and adjuvant temozolomide for glioblastoma. New Engl. J. Med. *352*, 987–996. https://doi.org/10.1056/NEJMoa043330.

2. Stupp, R., Hegi, M.E., Mason, W.P., van den Bent, M.J., Taphoorn, M.J., Janzer, R.C., Ludwin, S.K., Allgeier, A., Fisher, B., Belanger, K., et al. (2009). Effects of radiotherapy with concomitant and adjuvant temozolomide versus radiotherapy alone on survival in glioblastoma in a randomised phase III study: 5-year analysis of the EORTC-NCIC trial. Lancet Oncol. *10*, 459–466. https://doi.org/10.1016/S1470-2045(09)70025-7.

3. Grupp, S.A., Kalos, M., Barrett, D., Aplenc, R., Porter, D.L., Rheingold, S.R., Teachey, D.T., Chew, A., Hauck, B., Wright, J.F., et al. (2013). Chimeric antigen receptor-modified T cells for acute lymphoid leukemia. New Engl. J. Med. *368*, 1509–1518. https://doi.org/10.1056/NEJMoa1215134.

4. Porter, D.L., Levine, B.L., Kalos, M., Bagg, A., and June, C.H. (2011). Chimeric antigen receptor-modified T cells in chronic lymphoid leukemia. New Engl. J. Med. *365*, 725–733. https://doi.org/10.1056/NEJMoa1103849.

5. Abramson, J.S., McGree, B., Noyes, S., Plummer, S., Wong, C., Chen, Y.-B., Palmer, E., Albertson, T., Ferry, J.A., and Arrillaga-Romany, I.C. (2017). Anti-CD19 CAR T cells in CNS diffuse large-B-cell lymphoma. New Engl. J. Med. *377*, 783–784. https://doi.org/10.1056/NEJMc1704610.

6. Locke, F.L., Neelapu, S.S., Bartlett, N.L., Siddiqi, T., Chavez, J.C., Hosing, C.M., Ghobadi, A., Budde, L.E., Bot, A., Rossi, J.M., et al. (2017). Phase 1 results of ZUMA-1: a multicenter study of KTE-C19 anti-CD19 CAR T cell therapy in refractory aggressive lymphoma. Mol. Ther. : J. Am. Soc. Gene Ther. *25*, 285–295. https://doi.org/10.1016/j.ymthe.2016.10.020.

7. Kochenderfer, J.N., Dudley, M.E., Kassim, S.H., Somerville, R.P., Carpenter, R.O., Stetler-Stevenson, M., Yang, J.C., Phan, G.Q., Hughes, M.S., Sherry, R.M., et al. (2015). Chemotherapy-refractory diffuse large B-cell lymphoma and indolent B-cell malignancies can be effectively treated with autologous T cells expressing an anti-CD19 chimeric antigen receptor. J. Clin. Oncol. *33*, 540–549. https://doi.org/10.1200/jco.2014.56.2025.

8. Mount, C.W., Majzner, R.G., Sundaresh, S., Arnold, E.P., Kadapakkam, M., Haile, S., Labanieh, L., Hulleman, E., Woo, P.J., Rietberg, S.P., et al. (2018). Potent antitumor efficacy of anti-GD2 CAR T cells in H3-K27M(+) diffuse midline gliomas. Nat. Med. *24*, 572–579. https://doi.org/10.1038/s41591-018-0006-x.

9. Brown, C.E., Alizadeh, D., Starr, R., Weng, L., Wagner, J.R., Naranjo, A., Ostberg, J.R., Blanchard, M.S., Kilpatrick, J., Simpson, J., et al. (2016). Regression of glioblastoma after chimeric antigen receptor T-cell therapy. New Engl. J. Med. *375*, 2561–2569. https://doi.org/10.1056/NEJMoa1610497.

10. Morgan, R.A., Yang, J.C., Kitano, M., Dudley, M.E., Laurencot, C.M., and Rosenberg, S.A. (2010). Case report of a serious adverse event following the administration of T cells transduced with a chimeric antigen receptor recognizing ERBB2. Mol. Ther. *18*, 843–851. https://doi.org/10.1038/mt.2010.24.

11. Ahmed, N., Brawley, V.S., Hegde, M., Robertson, C., Ghazi, A., Gerken, C., Liu, E., Dakhova, O., Ashoori, A., Corder, A., et al. (2015). Human epidermal growth factor receptor 2 (HER2) -specific chimeric antigen receptor-modified T cells for the immunotherapy of HER2-positive sarcoma. J. Clin. Oncol. *33*, 1688–1696. https://doi.org/10.1200/jco.2014.58.0225.

12. Shiina, S., Ohno, M., Ohka, F., Kuramitsu, S., Yamamichi, A., Kato, A., Motomura, K., Tanahashi, K., Yamamoto, T., Watanabe, R., et al. (2016). CAR T cells targeting podoplanin reduce orthotopic glioblastomas in mouse brains. Cancer Immunol. Res. *4*, 259–268. https://doi.org/10.1158/2326-6066.cir-15-0060.

13. Krishnan, H., Rayes, J., Miyashita, T., Ishii, G., Retzbach, E.P., Sheehan, S.A., Takemoto, A., Chang, Y.W., Yoneda, K., Asai, J., et al. (2018). Podoplanin: an emerging cancer biomarker and therapeutic target. Cancer Sci. *109*, 1292–1299. https://doi.org/10.1111/cas.13580.

14. Mishima, K., Kato, Y., Kaneko, M.K., Nishikawa, R., Hirose, T., and Matsutani, M. (2006). Increased expression of podoplanin in malignant astrocytic tumors as a novel molecular marker of malignant progression. Acta Neuropathol. *111*, 483–488. https://doi.org/10.1007/s00401-006-0063-y.

15. Motomura, K., Natsume, A., Watanabe, R., Ito, I., Kato, Y., Momota, H., Nishikawa, R., Mishima, K., Nakasu, Y., Abe, T., et al. (2012). Immunohistochemical analysis-based proteomic subclassification of newly diagnosed glioblastomas. Cancer Sci. *103*, 1871–1879. https://doi.org/10.1111/j.1349-7006.2012.02377.x.

16. Verhaak, R.G., Hoadley, K.A., Purdom, E., Wang, V., Qi, Y., Wilkerson, M.D., Miller, C.R., Ding, L., Golub, T., Mesirov, J.P., et al. (2010). Integrated genomic analysis identifies clinically relevant subtypes of glioblastoma characterized by abnormalities in PDGFRA, IDH1, EGFR, and NF1. Cancer Cell *17*, 98–110. https://doi.org/10.1016/j.ccr.2009.12.020.

17. Chandramohan, V., Bao, X., Kato Kaneko, M., Kato, Y., Keir, S.T., Szafranski, S.E., Kuan, C.T., Pastan, I.H., and Bigner, D.D. (2013). Recombinant anti-podoplanin (NZ-1) immunotoxin for the treatment of malignant brain tumors. Int. J. Cancer *132*, 2339–2348. https://doi.org/10.1002/ijc.27919.

18. Schacht, V., Dadras, S.S., Johnson, L.A., Jackson, D.G., Hong, Y.K., and Detmar, M. (2005). Up-regulation of the lymphatic marker podoplanin, a mucin-type transmembrane glycoprotein, in human squamous cell carcinomas and germ cell tumors. Am. J. Pathol. *166*, 913–921. https://doi.org/10.1016/s0002-9440(10)62311-5.

19. Kahn, H.J., Bailey, D., and Marks, A. (2002). Monoclonal antibody D2-40, a new marker of lymphatic endothelium, reacts with Kaposi's sarcoma and a subset of angiosarcomas. Mod. Pathol. *15*, 434–440. https://doi.org/10.1038/modpathol.3880543.

20. Roy, S., Chu, A., Trojanowski, J.Q., and Zhang, P.J. (2005). D2-40, a novel monoclonal antibody against the M2A antigen as a marker to distinguish hemangioblastomas from renal cell carcinomas. Acta Neuropathol. *109*, 497–502. https://doi.org/10.1007/s00401-005-0999-3.

21. Kato, Y., and Kaneko, M.K. (2014). A cancer-specific monoclonal antibody recognizes the aberrantly glycosylated podoplanin. Sci. Rep. *4*, 5924. https://doi.org/10.1038/srep05924.

22. Fukuhara, H., Ino, Y., and Todo, T. (2016). Oncolytic virus therapy: a new era of cancer treatment at dawn. Cancer Sci. *107*, 1373–1379. https://doi.org/10.1111/cas.13027.

23. Desjardins, A., Gromeier, M., Herndon, J.E., 2nd, Beaubier, N., Bolognesi, D.P., Friedman, A.H., Friedman, H.S., McSherry, F., Muscat, A.M., Nair, S., et al. (2018). Recurrent glioblastoma treated with recombinant poliovirus. New Engl. J. Med. *379*, 150–161. https://doi.org/10.1056/NEJMoa1716435.

24. Kiyokawa, J., and Wakimoto, H. (2019). Preclinical and clinical development of oncolytic adenovirus for the treatment of malignant glioma. Oncolytic Virotherapy *8*, 27–37. https://doi.org/10.2147/ov.s196403.

25. Chiocca, E.A., and Rabkin, S.D. (2014). Oncolytic viruses and their application to cancer immunotherapy. Cancer Immunol. Res. *2*, 295–300. https://doi.org/10.1158/2326-6066.cir-14-0015.

26. Kim, M., Nitschke, M., Sennino, B., Murer, P., Schriver, B.J., Bell, A., Subramanian, A., McDonald, C.E., Wang, J., Cha, H., et al. (2018). Amplification of oncolytic

vaccinia virus widespread tumor cell killing by sunitinib through multiple mechanisms. Cancer Res. *78*, 922–937. https://doi.org/10.1158/0008-5472.can-15-3308.

27. Mineta, T., Rabkin, S.D., Yazaki, T., Hunter, W.D., and Martuza, R.L. (1995). Attenuated multi-mutated herpes simplex virus-1 for the treatment of malignant gliomas. Nat. Med. *1*, 938–943. https://doi.org/10.1038/nm0995-938.

28. Todo, T., Martuza, R.L., Rabkin, S.D., and Johnson, P.A. (2001). Oncolytic herpes simplex virus vector with enhanced MHC class I presentation and tumor cell killing. Proc. Natl. Acad. Sci. USA *98*, 6396–6401. https://doi.org/10.1073/pnas.101136398.

29. Ahmed, N., Brawley, V., Hegde, M., Bielamowicz, K., Kalra, M., Landi, D., Robertson, C., Gray, T.L., Diouf, O., Wakefield, A., et al. (2017). HER2-Specific chimeric antigen receptor-modified virus-specific T cells for progressive glioblastoma: a phase 1 dose-escalation trial. JAMA Oncol. *3*, 1094–1101. https://doi.org/10.1001/jamaoncol.2017.0184.

30. Choi, B.D., Curry, W.T., Carter, B.S., and Maus, M.V. (2018). Chimeric antigen receptor T-cell immunotherapy for glioblastoma: practical insights for neurosurgeons. Neurosurg. Focus *44*, E13. https://doi.org/10.3171/2018.2.focus17788.

31. Akhavan, D., Alizadeh, D., Wang, D., Weist, M.R., Shepphird, J.K., and Brown, C.E. (2019). CAR T cells for brain tumors: lessons learned and road ahead. Immunological Rev. *290*, 60–84. https://doi.org/10.1111/imr.12773.

32. Bagley, S.J., Desai, A.S., Linette, G.P., June, C.H., and O'Rourke, D.M. (2018). CAR T-cell therapy for glioblastoma: recent clinical advances and future challenges. Neuro-Oncology *20*, 1429–1438. https://doi.org/10.1093/neuonc/noy032.

33. Chuntova, P., Downey, K.M., Hegde, B., Almeida, N.D., and Okada, H. (2018). Genetically engineered T-cells for malignant glioma: overcoming the barriers to effective immunotherapy. Front. Immunol. *9*, 3062. https://doi.org/10.3389/fimmu.2018.03062.

34. Curran, K.J., Pegram, H.J., and Brentjens, R.J. (2012). Chimeric antigen receptors for T cell immunotherapy: current understanding and future directions. J. Gene Med. *14*, 405–415. https://doi.org/10.1002/jgm.2604.

35. Lamers, C.H., Sleijfer, S., van Steenbergen, S., van Elzakker, P., van Krimpen, B., Groot, C., Vulto, A., den Bakker, M., Oosterwijk, E., Debets, R., and Gratama, J.W. (2013). Treatment of metastatic renal cell carcinoma with CAIX CAR-engineered T cells: clinical evaluation and management of on-target toxicity. Mol. Ther. *21*, 904–912. https://doi.org/10.1038/mt.2013.17.

36. Linette, G.P., Stadtmauer, E.A., Maus, M.V., Rapoport, A.P., Levine, B.L., Emery, L., Litzky, L., Bagg, A., Carreno, B.M., Cimino, P.J., et al. (2013). Cardiovascular toxicity and titin cross-reactivity of affinity-enhanced T cells in myeloma and melanoma. Blood *122*, 863–871. https://doi.org/10.1182/blood-2013-03-490565.

37. Rosewell Shaw, A., and Suzuki, M. (2018). Oncolytic viruses partner with T-cell therapy for solid tumor treatment. Front. Immunol. *9*, 2103. https://doi.org/10.3389/fimmu.2018.02103.

38. Huang, J., Zheng, M., Zhang, Z., Tang, X., Chen, Y., Peng, A., Peng, X., Tong, A., and Zhou, L. (2021). Interleukin-7-loaded oncolytic adenovirus improves CAR-T cell therapy for glioblastoma. Cancer Immunol. Immunother. *70*, 2453–2465. https://doi.org/10.1007/s00262-021-02856-0.

39. McGrath, K., and Dotti, G. (2021). Combining oncolytic viruses with chimeric antigen receptor T cell therapy. Hum. Gene Ther. *32*, 150–157. https://doi.org/10.1089/hum.2020.278.

40. Nishio, N., Diaconu, I., Liu, H., Cerullo, V., Caruana, I., Hoyos, V., Bouchier-Hayes, L., Savoldo, B., and Dotti, G. (2014). Armed oncolytic virus enhances immune functions of chimeric antigen receptor-modified T cells in solid tumors. Cancer Res. *74*, 5195–5205. https://doi.org/10.1158/0008-5472.can-14-0697.

41. Tanoue, K., Rosewell Shaw, A., Watanabe, N., Porter, C., Rana, B., Gottschalk, S., Brenner, M., and Suzuki, M. (2017). Armed oncolytic adenovirus-expressing PD-L1 mini-body enhances antitumor effects of chimeric antigen receptor T cells in solid tumors. Cancer Res. *77*, 2040–2051. https://doi.org/10.1158/0008-5472.can-16-1577.

42. O'Rourke, D.M., Nasrallah, M.P., Desai, A., Melenhorst, J.J., Mansfield, K., Morrissette, J.J.D., Martinez-Lage, M., Brem, S., Maloney, E., Shen, A., et al. (2017). A single dose of peripherally infused EGFRvIII-directed CAR T cells mediates antigen loss and induces adaptive resistance in patients with recurrent glioblastoma. Sci. Translational Med. *9*. https://doi.org/10.1126/scitranslmed.aaa0984.

43. Ohno, M., Ohkuri, T., Kosaka, A., Tanahashi, K., June, C.H., Natsume, A., and Okada, H. (2013). Expression of miR-17-92 enhances anti-tumor activity of T-cells transduced with the anti-EGFRvIII chimeric antigen receptor in mice bearing human GBM xenografts. J. Immunother. Cancer *1*, 21. https://doi.org/10.1186/2051-1426-1-21.

44. Kato, Y., Vaidyanathan, G., Kaneko, M.K., Mishima, K., Srivastava, N., Chandramohan, V., Pegram, C., Keir, S.T., Kuan, C.T., Bigner, D.D., and Zalutsky, M.R. (2010). Evaluation of anti-podoplanin rat monoclonal antibody NZ-1 for targeting malignant gliomas. Nucl. Med. Biol. *37*, 785–794. https://doi.org/10.1016/j.nucmedbio.2010.03.010.

OMTO, Volume *26*

Supplemental information

Efficacy of cancer-specific anti-podoplanin CAR-T cells and oncolytic herpes virus G47Δ combination therapy against glioblastoma

Lushun Chalise, Akira Kato, Masasuke Ohno, Sachi Maeda, Akane Yamamichi, Shunichiro Kuramitsu, Satoshi Shiina, Hiromi Takahashi, Sachiko Ozone, Junya Yamaguchi, Yukinari Kato, Yumi Rockenbach, Atsushi Natsume, and Tomoki Todo

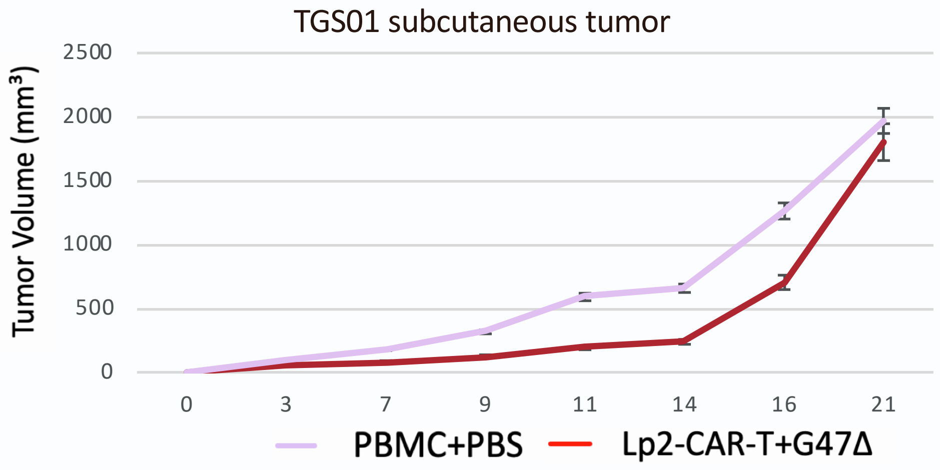

Figure S1. Against TGS01 subcutaneous tumors, the combination of Lp2-CAR-T cells and G47Δ slowed tumor growth for a short period, and it did not improve overall survival
